# Diuretic Potential of Whole Plant Extracts of *Pergularia daemia* (Forsk.)

**Published:** 2011

**Authors:** Vyas Bhavin, Vyas Ruchi, Santani DD

**Affiliations:** a *Maliba Pharmacy College, Gopal Vidyanagar, Tarsadi, India.*; b *Rofel, Shri G. M. Bilakhia College of Pharmacy, Vapi, India.*

**Keywords:** *Daemia extensa*, Diuretic activity, Diuretic action, Electrolyte

## Abstract

The whole plant, *Pergularia daemia *(Family: Asclepediaceae), was extracted with 50% alcohol and a fresh batch of the plant material was successively extracted with petroleum ether, ethyl acetate and *n*-butanol to determine its diuretic activity. The diuretic activity of the different extracts at a dose of 400 mg/Kg was assessed orally in rats with furosemide as a standard drug using Lipschitzs test. All extracts except the petroleum ether extract showed significant increase (p < 0.001) in urine output. Urinary electrolyte excretion was also affected by the extracts: the alcoholic, ethyl acetate and *n*-butanol extract caused an increase in the urinary excretion of sodium and potassium ions. These findings suggest that among the mentioned extracts, ethanolic has the maximum diuretic activity followed by *n*-butanol extract.

## Introduction

The plant *Pergularia daemia *(Asclepiadaceae) is known as “Uttaravaruni” in Sanskrit and “Utranajutuka” in Hindi. Traditionally the plant *P.daemia *was used as anthelmintic, laxative, antipyretic and expectorant, and was also used to treat infantile diarrhoea and malarial intermittent fevers and possesses stomachic, laxative and diuretic properties, useful in cough, biliousness and sore eyes (1-3). This plant has been reported to have anti-fertility (4), antidiabetic (5), wound healing (6), antibacterial (7), anti-inflammatory (8) and hepatoprotective activity (9).The plant extract is useful in uterine and menstrual disorder and in facilitating parturition. The juice of the leaves is used in asthma and applied in rheumatic swellings in combination with lime. Ethanol extract of aerial parts of *Pergularia daemia *(Forsk.) Chiov. was reported for anti-inflammatory, antipyretic, analgesic activity (9). Presence of triterpenes and saponins cardenolides and alkaloids were reported by Sathish *et al. *(11). Aanjaneyulu *et al. *(12) reported the presence of various triterpenes and steroidal compounds (3). In the present study, the ethanolic, pet ether, ethyl acetate and *n*-butanol extracts of *P. daemia *were screened for diuretic properties, to assess the medicinal potential of this plant.

## Experimental

*Extraction and fractionation*

The plant material was extracted by soxhlet with 50% alcohol and then concentrated. A fresh batch of the plant material was successively extracted with pet ether, ethyl acetate and *n*-butanol (in the increasing order of polarity) and was concentrated in the same order in a rotary evaporator at reduced pressure.

*Phytochemical analysis*

The extract and fractions was screened for various constituents (alkaloids, saponins, tannins, anthraquinones, sterol, flavonoids, terpenoids, glycosides, simple sugars) using standard protocol (13).

*Animals*

Wistar albino rats (180-250 g) and Swiss albino mice of either sex were acclimatized for 7 days under standard husbandry conditions, i.e. room temperature 35 ± 1°C, relative humidity 45-55% and light/dark cycle 12/12 h. The experimental protocols were approved by the Institutional Animal Ethical Committee (IAEC) of CPCSEA (Committee for the Purpose of Control and Supervision of Experiments on Animals).

*Acute toxicity test*

Acute oral toxicity study was performed as per OECD-423 guidelines (acute toxic class method) (14). Albino mice (n = 6) of either sex selected through random sampling technique was used for acute toxicity study. The animals were kept fasting for overnight providing only water, after which the extract (50% alcoholic extract) was administered orally at the dose level of 5 mg/Kg body weight by gastric intubation and observed for 14 days. If mortality is observed in 2 out of 3 animals, then the dose administered would be assigned as toxic dose. If mortality is observed in 1 animal, then the same dose would be repeated again to confirm the toxic dose. If mortality is not observed, the procedure would be repeated for further higher doses such as 50, 300 and 2000 mg/Kg body weight. According to the results of acute toxicity test, the doses were chosen for experiments.

*Diuretic activity*

The method described Wiebelhaus *et al. *(15) was employed, with modification, for the assessment of diuretic activity. Healthy albino rats of either sex (160-200 g) were divided into six groups of six animals each. They were fasted 18 h prior to the test, with free access to water. On the day of the experiment, animals were given 25 mL/Kg of body weight normal saline orally. Group I received vehicle (0.2 mL of 5% tween 80) and served as control group. Groups II, III, IV and V were treated with Standard drug (Furosemide 100 mg/Kg p.o.), ethanolic extract (400 mg/Kg), pet ether extract (400 mg/Kg), ethyl acetate extract (400 mg/Kg) and *n*-butanol extract (400 mg/Kg) respectively. All drugs/vehicle were administered orally (p.o.). Immediately after dosing, the rats were placed in the metabolic cages with special provision to collect faeces and urine. Animals were kept at room temperature of 35 ± 1°C throughout the experiment. Urine excreted for the next 5 h was collected and the total 5 h urine volume for each rat was compared with the volume of urine produced after the administration of normal saline.

The volume of urine excreted during 5 h for each animal in the group is expressed as the percent of the liquid (normal saline) administered. This percentage gives a measure of urinary excretion independent of the animal weight. The ratio of urinary excretion in the test group to urinary excretion in the control group is used as a measure of the diuretic action for the given dose of the drug. As the diuretic action is prone to variability, a parameter known as diuretic activity was calculated instead. To obtain the diuretic activity, the diuretic action of the extract is compared to that of the standard drug in the test group (16).

$\mathrm{Percentage of saline load excreted}=\frac{\mathrm{volum}\mathrm{of}\mathrm{urine}}{\mathrm{volum}\mathrm{of}\mathrm{saline}\mathrm{load}}\times 100$

$\mathrm{Urinary}\mathrm{excretion}=\frac{\mathrm{total of urinary output}}{\mathrm{total liquid administered}}$

$\mathrm{Diuretic action}=\frac{\mathrm{urinary excretion of treated group}}{\mathrm{urinary excretion of control group}}$

$\mathrm{Diuretic activity}=\frac{\mathrm{diuretic action of test drug}}{\mathrm{diuretic action of standard drug}}$

The parameters taken to study were pH, Na^+^, K^+^ and Cl^-^ concentration in urine. Urine samples were analyzed thereafter for Na^+^ and K^+^ concentration by flame photometric method while Cl^-^ concentration will be determined titrimetrically and the results were reported as mean ± SEM.

*Statistical analysis*

The data were expressed as mean ± SEM. The data of diuretic activity were analyzed by one-way analysis of variance (ANOVA) followed by “Dunnett’s test.” p-value < 0.05 was considered statistically significant.

## Results and Discussion

Result of preliminary phytochemical analysis conducted on the different extracts *i.e. *alcoholic (50%), petroleum ether, ethyl acetate and *n*-butanol extract of *Pergularia daemia *indicates presence of flavonoids, steroids, alkaloids, triterpens and saponin. Acute toxicity studies showed that the alcoholic extracts did not cause any mortality up to 2000 mg/Kg and were considered as safe (17).

This study examined the diuretic potential of Pergularia daemia. The results showed that all the extracts, except petroleum ether extract, increases urine output, which is expressed as the percentage of saline load excreted, as compared to control group (Table 1). 

**Table 1:** Effect of various extracts of *Pergularia daemia *on the percent excretion of administered saline load in the rat

**Treatment**	**Dose (mg/Kg)**	**Percentage of saline load excreted**	**Diuretic action**
Normal Saline	25 mL/Kg	38.44 ± 4.36	1
Standard (Furosemide)	100	84.22 ± 6.78**	2.19
Alcoholic extract	400	78.44 ± 7.36**	2.04
Pet ether extract	400	42 ± 4.63	1.09
Ethyl acetate extract	400	63.77 ± 5.94*	1.66
*n*-butanol extract	400	66.44 ± 5.52**	1.73

Each value represents the mean + SEM. of six rats.* p < 0.05; **p < 0.001

The diuretic action of furosemide, alcoholic extract, pet ether extract, ethyl acetate extract and *n*-butanol extract was found to be 2.19, 2.04, 1.09, 1.66 and 1.73 respectively as compared with 1 of control group (Table. 1). Diuretic activity of alcoholic extract was found to be highest 0.93 followed by 0.79 of *n*-butanol and 0.76 of ethyl acetate extract (Table 2).

**Table 2:** Diuretic activity of different extracts of *Pergularia daemia*

**Treatment**	**Diuretic activity**
Alcoholic extract	0.93
Pet ether extract	0.49
Ethyl acetate extract	0.76
*n*-butanol extract	0.79

The results show that alcoholic extract, ethyl acetate extract and *n*-butanol extract of *Pergularia daemia *(Table 3) affects urinary electrolyte. All the extracts were not accompanied with reduction in urinary K^+^ level. In addition there was no alkalization of urine. These data indicate that they are not acting as potassium sparing diuretics (18, 19). The extracts were also unlikely to be acting as thiazide diuretics: these only increase urinary K^+^ level and alter the urinary Na^+^/K^+^ ratio (17). But in this study both urinary Na^+^ and K^+^ level were increased without any alteration in Na^+^/K^+^ ratio.

**Table 3:** Effect of different extracts of *Pergularia daemia *on electrolyte excretion and pH in the rat

**Treatment**	**Dose (mg/Kg)**	**Electrolyte Concentration (meq/L)**				**pH**
		Na^+^	K^+^	Cl^-^	Na^+^/ K^+ ^ratio	
Normal Saline	25 mL/Kg	84.52 ± 0.48	55.71 ± 1.23	92.70 ± 1.06	1.51	
Standard (Furosemide)	100	139.11 ± 0.61*	89.42 ± 0.93*	93.13 ± 0.57	1.55	6.3 ± 0.12
Alcoholic extract	400	138.22 ± 1.05*	82.93 ± 0.82*	93.49 ± 0.96	1.59	6.4 ± 0.16
Pet ether extract	400	87.34 ± 1.32	57.23 ± 1.03	92.56 ± 0.56	1.52	6.9 ± 0.15
Ethyl acetate extract	400	121.36 ± 1.5*	79.39 ± 0.22*	91.85 ± 0.41	1.54	6.5 ± 0.15
*n*-butanol extract	400	131.89 ± 0.98*	85.74 ± 0.67*	91.54 ± 0.87	1.54	6.5 ± 0.12

Each value represents the mean + SEM. of six rats.* p < 0.001

In contrast, the diuresis induced by alcoholic, ethyl acetate and *n*-butanol extract of *Pergularia daemia*, was similar to that of furosemide and accompanied by marked increases in both urinary Na^+^ and K^+^ level. Further the urine was slightly acidified (Table 3). These characteristics strongly suggest these extracts are acting as loop diuretic. Loop diuretics inhibit the Na^+^/K^+^/Cl^-^ co-transporter system in the thick ascending loop of nephron, thereby increasing natriuresis and kaleuresis (18, 19) and also cause acidification of urine (18, 20).

In conclusion, the extract might be able to act as loop diuretic and for understanding its mechanism of action more experiments is required. The diuretic activity of alcoholic, ethyl acetate and *n*-butanol extracts may be attributed to presence of alkaloids, flavonoid or steroids.
